# COVID-19 IN LATIN AMERICA: CONFLICTS, RESISTANCES AND INEQUALITIES

**DOI:** 10.1590/S0104-59702023000100028

**Published:** 2023-08-14

**Authors:** Claudia Agostoni, Karina Ramacciotti, Carlos Henrique Paiva, Marcos Cueto

**Affiliations:** i Instituto de Investigaciones Históricas/Universidad Nacional Autónoma de México. Ciudad de México – México; ii Universidad Nacional de Quilmes/Conicet. Ciudad Autónoma de Buenos Aires – Buenos Aires – Argentina; Conicet, Ciudad Autónoma de Buenos Aires, Buenos Aires, Argentina; iii Observatório História e Saúde/Casa de Oswaldo Cruz/Fiocruz. Rio de Janeiro – RJ – Brasil; iv Casa de Oswaldo Cruz/Fiocruz. Rio de Janeiro – RJ – Brasil

The covid-19 pandemic intensified deep social inequalities in developing countries, revealed inadequate official responses, exposed the geopolitical inequities that separate rich and developing countries, and gave rise to heated debates about the human body, disease, prevention and the state of health systems. In each of these debates, science and politics converged and were the subject of fierce discussions that, in some countries, further divided society. The pandemic also revealed the coexistence of long-standing precariousness, tensions and resiliencies in health systems. In this sense, it is worth mentioning the inequality in the distribution of supplies, initially masks and personal protective equipment and, later on, vaccines, as well as the inequitable, ineffective or non-existent presence and distribution of trained human resources with decent working conditions. Likewise, analysts called attention to the existence of a pattern of state response to the pandemic, initially focused almost exclusively on the hospital, to the detriment of primary health care (Giovanella et al., 2021), whose process of institutional consolidation, its territorial capillarity and its importance in disease control existed before the pandemic in countries such as Brazil (Paiva, Pires-Alves, 2021).

Latin America was also the epicenter of covid-19 for much of 2020 and 2021, both for the highest number of cases and highest fatality rate, as well as for hosting the highest percentage of infected people in relation to its population globally. The result indicates that it is one of the world’s most socially unequal and still biomedically dependent regions. The region was also the scene of extreme cases. These include the abandonment of the sick and corpses in the streets of Guayaquil, Ecuador, and the mass burials in Manaus, Brazil, in early 2020. Government responses marked by negligence and genocide as in Jair Bolsonaro’s Brazil; collapsed hospitals and scarce resources stretched to their limits; frustrated attempts to counterpose public health to the economy; strenuous efforts against adversity by health professionals, patients’ families and activists; the glorification of magical but ineffective remedies such as chloroquine; corruption in the purchase of vaccines; and multiple situations in which health personnel were first glorified and then subjected to violence as vehicles of contagion. With particular intensity there was a collapse of human resources in the countries of the region that revealed a history of negligence and minimization of this central human element of health care and prevention ( Agostoni, 2021 ; Ramacciotti, 2023 ).

The purpose of this supplement of *História, Ciências, Saúde – Manguinhos* is to gather original and relevant studies that, based on covid-19, establish a contextualized dialogue between the past and the present. Therefore, the articles discuss how the complex relationship between science, society and politics is magnified in health disasters; recording the challenges of health workers; identifying parallels and divergences in the main problems mentioned above, and claiming the importance of a comparative historical perspective to analyze and understand epidemics and contemporary public health. They also present detailed reflections on the interactions between science, health and society in crisis situations, which will be of interest to historians and other social scientists who may wish to deepen their understanding and historical analysis of the pandemic in the future.

These works have an additional originality. They were carried out at the same time as the pandemic was developing and intensifying, a time when individual, collective and institutional reactions and responses were uncertain, divergent and contradictory. Thus, another element addressed by the articles is the multifaceted social and institutional perception of the new disease, reaffirming that the coronavirus disease is much more than a biomedical event. Therefore, the representations, practices, imaginaries and debates related to the etiology, spread and forms of containment of the new disease are part of the studies presented here.

Measures of social-distancing and isolation to prevent the spread of the coronavirus affected social and economic life and research activities. With libraries and archives closed, social science work was interrupted. Undoubtedly, the digitalization of some archives, whether open access or private, allowed some researchers to sustain their lines of work. However, for those who had been working on different historical and sociological aspects of the social, political and cultural processes related to get sick, being cured and cared, many of the questions and theoretical frameworks used for other societies and periods were put under discussion as of March 2020 (Campos, Perdiguero-Gil, Bueno, 2020; Hochman, Birn, 2021). Likewise, the coronavirus generated a special section in the *História, Ciências, Saúde – Manguinhos* journal blog, where prominent historians of the region published reflections on the pandemic. Subsequently, elaborated versions of these texts were published in the journal. The journal also hosted other works, including an important debate in which some of the guest editors of this supplement participated (Agostoni, Ramacciotti, Lopes, 2022).

The memory of other plagues and diseases as well as the serious asymmetries in the global distribution of medical resources were once again present, and it is precisely this present that renewed questions about the past and invited us to understand how other societies, in other times, had faced similar phenomena. For this reason, and as this supplement shows, we collected, in real time, data from interviews with different social actors, periodical press, surveys, and analysis of regulations to put them in dialogue with the theoretical frameworks of the social sciences. Many of these ways of gathering information were different from the usual ones; for example, most of the interviews were conducted in virtual format, which undoubtedly opens new ways of thinking about methodology in exceptional times and invites us to reflect on the place of history and social sciences to provide answers to critical situations and renews research agendas.

We hope that these studies, which cover some of the countries of the region and the issues that went through the pandemic, will be a contribution to understanding how covid-19 marked Latin America.
